# The impact of professional music performance competence on performance anxiety: the mediating role of psychological risk and moderating role of psychological resilience

**DOI:** 10.3389/fpsyg.2025.1565215

**Published:** 2025-03-06

**Authors:** Yongde Yang, Peng Lei, Zumin Huang, Hongle Yu, Huoyin Zhang

**Affiliations:** ^1^School of Music and Performance, Yibin University, Yibin, China; ^2^China Center for Behavioral Economics and Finance, Southwestern University of Economics and Finance, Chengdu, China; ^3^Faculty of Pedagogy and Psychology, Southwest Minzu University, Chengdu, China; ^4^School of Psychology, Sichuan Normal University, Chengdu, China; ^5^School of Psychology, Shenzhen University, Shenzhen, China

**Keywords:** music performance anxiety, professional competence, psychological risk, psychological resilience, moderated mediation effect

## Abstract

**Introduction:**

Music performance anxiety (MPA) significantly impacts musicians’ wellbeing and career development, yet the complex interplay between professional competence, psychological risk, and resilience in MPA formation remains understudied.

**Methods:**

This research investigated these mechanisms through a moderated mediation model among 609 music major students (70.9% female; *M* age = 20.96 years, SD = 4.52). Participants completed a comprehensive assessment battery including the Professional Questionnaire for Musicians (PQM), Psychological Risk Questionnaire for Musicians (PRQM), Kenny Music Performance Anxiety Inventory (K-MPAI), and Connor-Davidson Resilience Scale (CD-RISC). Data were analyzed using SPSS 26.0 and PROCESS macro (Model 7).

**Results:**

Analysis revealed significant negative correlations between professional competence and both performance anxiety (*r* = −0.252, *p* < 0.01) and psychological risk (*r* = −0.448, *p* < 0.01). Psychological risk significantly mediated the relationship between professional competence and performance anxiety (indirect effect = −0.171, 95% CI [−0.243, −0.099]). Notably, psychological resilience moderated this indirect effect (moderation effect = 0.188, 95% CI [0.092, 0.284]), with the relationship being strongest at low resilience levels (*β* = −0.881, *p* < 0.001) and weakest at high resilience levels (*β* = −0.693, *p* < 0.001).

**Discussion:**

These findings validate a complex mechanism wherein professional competence influences performance anxiety through psychological risk, with psychological resilience serving as a crucial moderating factor. The results emphasize the importance of developing multidimensional intervention strategies and provide empirically-grounded guidance for music education practice, suggesting that enhancing both professional competence and psychological resilience may effectively reduce performance anxiety through the mitigation of psychological risk.

## 1 Introduction

### 1.1 Problem statement

Music performance anxiety is a pervasive phenomenon affecting both students and professional musicians. Research indicates that over 60% of professional musicians have experienced significant performance anxiety, with even higher rates among music students (Kenny and Osborne, 2006; Papageorgi, 2022). This anxiety not only impacts performance quality but can also hinder career development, affect physical and mental health, and even lead some talented musicians to prematurely abandon their careers. While moderate anxiety may enhance performance, excessive anxiety often impairs the naturalness of musical expression and technical fluency, creating a vicious cycle (Papageorgi et al., 2013; de Lima et al., 2024).

Performance experiences, particularly negative ones, play a crucial role in the development of anxiety. A single adverse performance experience can leave a lasting psychological impact, altering one’s cognitive appraisal of future performance situations (Parncutt and McPherson, 2002). Intriguingly, research has found that even technically accomplished professional musicians may continue to struggle with performance anxiety due to early negative experiences. This suggests that merely enhancing professional competence is insufficient to fully resolve anxiety; greater attention must be paid to why individuals exhibit different psychological responses and adaptation levels when facing similar pressures (McPherson et al., 2019).

Existing research exploring the causes of music performance anxiety has largely focused on personality traits, situational characteristics, or social support factors. However, there remains a lack of systematic investigation into the interactive mechanisms among professional competence, psychological risk assessment, and individual psychological traits (such as psychological resilience). In today’s increasingly competitive music education and performance environment, effectively preventing and intervening in performance anxiety has become an urgent practical issue. Based on this, the present study aims to explore three core questions: How does professional competence influence performance anxiety? What role does psychological risk assessment play in this process? Can psychological resilience moderate this influence process? Answering these questions holds not only theoretical significance but will also provide valuable insights for music education practice and anxiety intervention.

### 1.2 Literature review

Music performance anxiety research has evolved significantly, with recent studies focusing on the intricate relationships among professional competence, cognitive risk assessment, and psychological resilience. This review synthesizes current understanding of these interconnected domains while identifying crucial research gaps.

The cognitive appraisal framework remains fundamental in understanding MPA, particularly through its emphasis on primary (threat assessment) and secondary (coping resource assessment) appraisal processes (Lazarus and Folkman, 1984). Recent research has significantly advanced this understanding, with identifying distinct MPA patterns across performance phases (Spahn et al., 2021), and establishing clear relationships between MPA and other anxiety manifestations (Dobos et al., 2019). Contemporary research has particularly illuminated the role of cognitive processes in MPA. Kaleńska-Rodzaj’s (2020) analysis of pre-performance emotions has demonstrated how cognitive appraisal directly influences anxiety management strategies. This understanding has been further enriched by investigation of flow states in relation to MPA (Cohen and Bodner, 2021), establishing the mediating role of cognitive processes in the anxiety-performance relationship.

Psychological risk assessment has emerged as a crucial mediating factor in MPA development. Lupiáñez et al. (2022) identified key predictive patterns in conservatory students, while Arbinaga (2023) demonstrated systematic variations in catastrophizing and somatic complaints across different performer groups. These findings extend Papageorgi et al.’s (2007) conceptual framework, particularly regarding the influence of subjective risk assessment on anxiety manifestation.

Recent investigations into psychological resilience have revealed promising intervention pathways. Faur et al.’s (2023) meta-analysis has demonstrated the efficacy of resilience-building approaches in anxiety reduction, while Jiang and Tong (2024) have illuminated how psychological capital influences MPA through self-esteem mechanisms. Cross-cultural perspectives have further enriched this understanding, with studies by Butković et al. (2022) and Gök and Yalçınkaya-Alkar (2024) revealing both universal and culturally specific aspects of anxiety manifestation.

The pedagogical dimension has received increased attention through systematic investigations of teaching approaches (Mazzarolo et al., 2023) and early intervention strategies (Bersh, 2022). Recent integrative researches have emphasized the necessity of comprehensive approaches incorporating both individual differences and contextual factors (Guyon et al., 2020; Martins et al., 2024).

Despite these advances, critical research gaps persist, particularly regarding: (1) the interactive effects among professional competence, psychological risk, and resilience; (2) systematic validation of influence mechanisms across diverse performance contexts; (3) comprehensive cross-cultural comparisons of MPA manifestation and management.

### 1.3 Research hypotheses

#### 1.3.1 The impact of professional competence on performance anxiety

According to cognitive appraisal theory (Lazarus and Folkman, 1984), professional competence, as an individual’s core coping resource, directly influences the secondary appraisal process. Higher professional competence enables individuals to make more positive resource assessments when facing performance tasks, thereby reducing anxiety levels. Additionally, based on the competence-demand matching theory, optimal psychological states occur when an individual’s professional competence matches performance requirements. Task demands that exceed competence levels lead to increased anxiety, while sufficient professional competence can reduce this imbalance (Burin and Osório, 2017). Third, from the perspective of attentional resource theory, higher professional competence increases skill automation, releasing more cognitive resources for positive performance goals rather than negative self-focus. Empirical research provides strong support for these theoretical mechanisms (Buma et al., 2015). González et al. (2018) found a significant negative correlation between professional competence and performance anxiety. Longitudinal studies have also confirmed the stability of this relationship, as Papageorgi et al. (2007) discovered through a two-year tracking study that improvements in professional competence significantly predicted reduced anxiety. Moreover, this influence is context-specific. Williamon and Thompson (2006) demonstrated that the negative correlation between professional competence and anxiety is more pronounced in high-difficulty performance tasks. Based on the above theoretical analysis and empirical evidence, this study proposes H1: Professional competence has a significant negative effect on performance anxiety.

#### 1.3.2 The mediating role of psychological risk

The mechanism by which professional competence influences psychological risk assessment can be understood from multiple theoretical perspectives. First, based on Bandura’s (1977) self-efficacy theory, professional competence alters subjective risk assessment by influencing individual efficacy expectations. Salmon (1990) applied this theory to music performance, finding that skill level directly affects performers’ cognitive assessment of threats. Second, from cognitive resource theory, Steptoe and Fidler (1987) noted that higher professional competence allows performers to allocate more attentional resources to positive performance goals rather than potential risks. Valentine (2002) revealed three key pathways through which professional competence influences psychological risk: (1) skill proficiency directly affects error risk assessment; (2) accumulated performance experience influences situational control perception; (3) professional training enhances coping strategy reserves. Osborne and Kenny’s (2008) longitudinal study (*N* = 410) further confirmed that increased professional competence significantly reduces performers’ concerns about negative evaluation (*β* = −0.43, *p* < 0.001).

As a cognitive antecedent of anxiety, the mediating role of psychological risk has been widely validated. Craske and Craig’s (1984) early experimental research first confirmed this mediating effect. Wilson (2002), through a study of 120 professional musicians, found that risk assessment could explain 47% of the variance in the relationship between professional competence and anxiety. Kenny et al. (2004) additionally found that this mediating effect remained stable across different performance types (solo vs. ensemble). Papageorgi et al.'s (2013) cross-cultural research further supported the universality of this mediating mechanism. Notably, LeBlanc et al. (1997) indicated that the mediating role of psychological risk might be moderated by performance context characteristics. This view was supported in Kobori et al.’s (2011) research, which found that risk assessment’s mediating effect was more significant in high-stakes performances. Based on the above theoretical analysis and empirical evidence, this study proposes H2: Psychological risk mediates the relationship between professional competence and performance anxiety.

#### 1.3.3 The moderating role of psychological resilience

As an important psychological resource, the theoretical foundation for the moderating role of psychological resilience can be traced back to Werner’s (1995) adaptive development model. In the field of music performance, Steptoe (1989) first proposed that psychological resilience might moderate anxiety generation through its influence on cognitive appraisal processes. This view aligns with Barlow’s (2000) vulnerability-stress model, which emphasizes the moderating role of individual traits in stress response processes. Martin and Marsh’s (2008) research revealed the core operational mechanism of psychological resilience in performance contexts. They found that individuals with higher psychological resilience typically demonstrate stronger cognitive reappraisal abilities, enabling them to view potential threats as challenges. This cognitive transformation ability directly influences the risk assessment process, thereby moderating anxiety generation. Fletcher and Sarkar (2012), through qualitative research on excellent performers, further confirmed these findings and emphasized that the protective function of psychological resilience is particularly crucial in high-pressure situations.

In terms of empirical research, MacNamara et al.’s (2010) tracking study additionally found that the moderating effect of psychological resilience on the relationship between professional competence and risk assessment remains stable throughout long-term career development. Notably, Kenny and Ackermann’s (2015) study of professional musicians confirmed the continuous protective effect of psychological resilience across different stages of professional development. Cheung et al.’s (2019) cross-cultural research further validated the moderating effect of psychological resilience, finding that this effect exists across different cultural backgrounds, though its intensity may vary with cultural characteristics. Yoshie et al. (2009) provided multi-level empirical support for the moderating role of psychological resilience through research methods combining physiological indicators and behavioral data. These findings collectively indicate that psychological resilience, as an important individual characteristic, can significantly influence how professional competence affects performance anxiety through risk assessment. Based on the above theoretical analysis and empirical evidence, this study proposes H3: Psychological resilience moderates the mediating pathway through which professional competence influences performance anxiety via psychological risk, specifically, the higher the psychological resilience, the weaker the mediating effect.

**Figure 1:**
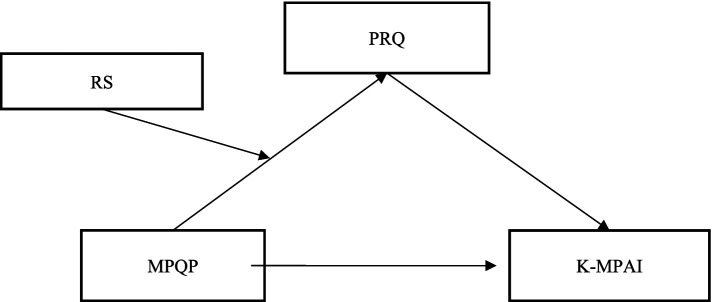
Conceptual model.

Based on the above theoretical analysis and empirical research, this study constructs a moderated mediation model to explain the formation mechanism of music performance anxiety. This model integrates the direct effect of professional competence, the mediating effect of psychological risk, and the moderating effect of psychological resilience, revealing the complex interactive relationships among these variables. Specifically, this study proposes that professional competence may influence performance anxiety through two pathways: first, a direct influence pathway, where higher professional competence leads to lower performance anxiety levels (H1); second, an indirect influence pathway, where professional competence reduces performance anxiety by lowering psychological risk assessment (H2). This mediating effect highlights the core role of cognitive appraisal in anxiety generation. Meanwhile, considering the importance of individual differences, this study introduces psychological resilience as a moderating variable, hypothesizing that it can moderate the mediating pathway through which professional competence influences performance anxiety via psychological risk (H3). This integrated model not only reflects the multiple mechanisms of performance anxiety formation but also demonstrates the dynamic interactive relationships among individual traits, cognitive appraisal, and emotional experience. The proposal of this model contributes to a more comprehensive understanding of the mechanisms underlying music performance anxiety and provides a theoretical foundation for developing subsequent intervention strategies (Figure 1).

This study makes several significant contributions to the field of music performance anxiety research. First, it advances theoretical understanding by integrating professional competence, psychological risk, and psychological resilience into a unified framework, moving beyond traditional single-factor approaches to reveal complex interaction mechanisms in performance anxiety formation. Second, it provides empirical validation of psychological risk’s mediating role and psychological resilience’s moderating function, offering new insights into how individual differences influence anxiety responses in performance contexts. Third, methodologically, the study employs sophisticated statistical techniques to test a moderated mediation model, providing a more nuanced understanding of variable relationships than previous correlational studies. Fourth, it bridges theoretical research with practical applications by offering evidence-based recommendations for music education reform and anxiety intervention strategies. Finally, while most existing research has focused on Western contexts, this study extends the investigation to a different cultural setting, contributing to the cross-cultural understanding of performance anxiety mechanisms. These contributions not only enrich the theoretical framework of performance anxiety research but also provide valuable guidance for educational practice and psychological intervention in music performance contexts.

## 2 Methodology

### 2.1 Participants and procedures

This investigation employed a systematic sampling approach at three major conservatories, targeting university students majoring in music. Following institutional review board approval from the Ethics Review Committee of the Sichuan Psychological Association and adherence to Declaration of Helsinki guidelines, the research team secured permissions from respective music departments. Standardized recruitment packages, containing study information and consent documentation, were distributed through departmental administrators to ensure procedural consistency and voluntary participation.

From an initial pool of 650 respondents, 609 valid questionnaires were retained after applying exclusion criteria (insufficient performance experience, incomplete responses, invalid response patterns, or withdrawn consent). The final sample demonstrated a gender distribution of 70.9% females (*n* = 432) and 29.1% males (*n* = 177), with participants ranging in age from 18 to 58 years (*M* = 20.96, SD = 4.52). Age distribution analysis revealed a concentration in the 18–21 year range, with 20-year-olds comprising the largest cohort (30.5%, *n* = 186), followed by 19-year-olds (20.9%, *n* = 127), 18-year-olds (18.1%, *n* = 110), and 21-year-olds (12.3%, *n* = 75).

The sample composition reflected diverse musical specializations, comprising 257 vocal majors (42.2%), 97 instrumental majors (15.9%), and 255 participants from related disciplines (41.9%). While vocal and instrumental majors had undergone systematic conservatory training, participants from related disciplines demonstrated continuous music engagement through interdisciplinary programs such as music education and music therapy. Performance proficiency assessment indicated that 87.2% (*n* = 531) of participants demonstrated smooth or proficient execution in their respective areas, with daily practice commitments predominantly ranging from 1 to 3 h (93.4%, *n* = 569).

Data collection spanned July through October 2024, utilizing both digital and traditional administration methods. The online component employed a professional survey platform, while in-person administration occurred in controlled classroom settings. Data quality assurance protocols included strategically placed attention check items, completion time monitoring, response pattern analysis, and reversed item consistency verification. This methodological framework yielded a sample size exceeding the priori power analysis requirements (Monte Carlo Power Analysis for Indirect Effects: *α* = 0.05, power = 0.90, *N* = 250), ensuring robust statistical power for hypothesis testing.

The resultant participant pool exhibited balanced representation across performance contexts and proficiency levels, supporting generalizability within formal music education settings. This systematic approach to sampling and data collection, combined with comprehensive inclusion criteria and rigorous validation procedures, established a methodologically sound foundation for investigating music performance anxiety within the target population. The sample’s distribution characteristics affirmed its representativeness regarding professional background, training commitment, and skill development levels.

### 2.2 Research instruments

#### 2.2.1 Performance quality measure for musicians (PQM)

The PQM, developed by McPherson and McCormick (2006), is a comprehensive tool for assessing musicians’ professional competence levels. The scale consists of 30 items evaluating four core dimensions: technical ability (10 items, e.g., “pitch control,” “rhythmic accuracy”), musical expressiveness (8 items, e.g., “sound quality,” “tonal variation”), stage presence (7 items, e.g., “stage manner,” “audience interaction”), and professional attributes (5 items, e.g., “rehearsal discipline,” “professional attitude”). It employs a 7-point rating scale (1 = “very poor” to 7 = “excellent”), with total scores ranging from 30 to 210. The scale demonstrates excellent psychometric properties, with an overall internal consistency coefficient of 0.92, and Cronbach’s *α* coefficients of 0.88, 0.86, 0.84, and 0.83 for the four dimensions, respectively. Confirmatory factor analysis supports its four-factor structure (CFI = 0.93, TLI = 0.91, RMSEA = 0.056). The scale has been applied and validated across multiple countries and regions, demonstrating good cross-cultural applicability.

#### 2.2.2 Performance risk questionnaire for musicians (PRQM)

The PRQM, developed by Papageorgi (2007), measures musicians’ subjective assessment of performance risks. The scale contains 20 items across three dimensions: task difficulty perception (8 items, e.g., “This piece is technically demanding for me”), failure consequence evaluation (7 items, e.g., “Performance mistakes would seriously affect my reputation”), and lack of control (5 items, e.g., “I feel unable to fully control the performance process”). It uses a 7-point Likert scale (1 = “strongly disagree” to 7 = “strongly agree”), with total scores ranging from 20 to 140. The scale demonstrates stable factor structure across different samples (CFI = 0.94, RMSEA = 0.052), with internal consistency coefficients of 0.85, 0.83, and 0.81 for each dimension, and 0.89 for the total scale.

#### 2.2.3 Kenny music performance anxiety inventory (KMPAI)

The KMPAI, developed by Kenny (2009) based on Barlow’s anxiety theory, is a professional tool for assessing music performance anxiety. The scale consists of 40 items evaluating three dimensions: cognitive (e.g., catastrophic thinking, worry), somatic (e.g., increased heart rate, sweating palms), and behavioral (e.g., avoidance, defense) manifestations of performance anxiety. It employs a 7-point scoring system (0 = “strongly disagree” to 6 = “strongly agree”), with total scores ranging from 0 to 240. The scale demonstrates excellent psychometric properties with an internal consistency coefficient of 0.94. Exploratory and confirmatory factor analyses support its three-factor structure (CFI = 0.95, RMSEA = 0.048). The scale has been translated into 12 languages and widely used in international research, demonstrating good cross-cultural applicability and diagnostic validity.

#### 2.2.4 Connor–Davidson resilience scale (CD-RISC)

The CD-RISC, developed by Connor and Davidson (2003), is an authoritative tool for assessing individual psychological resilience. The scale contains 25 items evaluating five dimensions: personal competence and tenacity (8 items), positive acceptance of change (7 items), secure relationships (4 items), control (3 items), and spiritual influences (3 items). It uses a 5-point scoring system (0 = “not true at all” to 4 = “true nearly all the time”), with total scores ranging from 0 to 100. The scale demonstrates a stable five-factor structure (CFI = 0.93, RMSEA = 0.049), with an overall internal consistency coefficient of 0.89, and dimensional coefficients ranging from 0.73 to 0.85. The scale has been widely applied across educational, medical, sports, and other fields, demonstrating good ecological validity.

### 2.3 Data analysis strategy

SPSS 26.0 was used for descriptive statistical analysis, including means and standard deviations of variables, and Pearson correlation coefficients were employed to examine relationships between variables. Additionally, Harman’s single-factor test was conducted to examine common method bias. In this test, all measurement items underwent unrotated exploratory factor analysis, where a common method bias is considered non-significant if the first factor explains less than 40% of the variance (Podsakoff et al., 2003). In this study, the first factor explained 32.8% of the variance, indicating no significant common method bias.

For testing mediation and moderated mediation effects, analyses were conducted using Hayes (2017) PROCESS macro. Specifically, Model 4 was used to test mediation effects, with 5,000 bootstrap resamples and 95% confidence intervals to determine the significance of mediation effects. Model 7 was employed to test moderated mediation effects, first examining the significance of moderation effects and plotting simple slopes, then calculating conditional indirect effects at different levels of the moderating variable (M ± 1SD), and finally computing the index of moderated mediation (Hayes, 2015). Following Zhao et al.’s (2010) recommendations, the specific type of mediation was determined by examining the significance and direction of both direct and indirect effects.

## 3 Results

### 3.1 Descriptive statistics

To explore the basic characteristics and relationships among research variables, descriptive statistical analysis and correlation analysis were conducted for music performance professional competence, psychological risk, performance anxiety, and psychological resilience (see Table 1). Results showed that the mean score for the Music Performance Quality Profile (MPQP) was 46.13 (SD = 8.49), the Psychological Risk Questionnaire (PRQ) was 104.05 (SD = 16.24), the Kenny Music Performance Anxiety Inventory (K-MPAI) was 124.83 (SD = 28.55), and the Resilience Scale (RS) was 56.09 (SD = 15.10). Correlation analysis revealed that music performance professional competence was significantly negatively correlated with psychological risk (*r* = −0.448, *p* < 0.01), performance anxiety (*r* = −0.252, *p* < 0.01), and psychological resilience (*r* = −0.140, *p* < 0.01). Psychological risk showed significant positive correlations with performance anxiety (*r* = 0.383, *p* < 0.01) and psychological resilience (*r* = 0.161, *p* < 0.01). Performance anxiety was significantly negatively correlated with psychological resilience (*r* = −0.182, *p* < 0.01). These correlation results provided preliminary support for subsequent mediation and moderation analyses.

**Table 1:** Descriptive statistics and correlation coefficients of variables (*N* = 609).

Variable	*M*	SD	1	2	3
1. MPQP	46.13	8.49	–		
2. PRQ	104.05	16.24	−0.448**	–	
3. K-MPAI	124.83	28.55	−0.252**	0.383**	–
4. RS	56.09	15.1	−0.140**	0.161**	−0.182**

MPQP, Music Performance Quality Questionnaire; PRQ, Psychological Risk Questionnaire; K-MPAI, Kenny Music Performance Anxiety Inventory; RS, Resilience Scale. **p < 0.01.

### 3.2 Moderated mediation effect analysis

The PROCESS macro (Model 7) developed by Hayes (2017) was used to test the moderated mediation model. Specifically, this study examined the mediating role of psychological risk in the relationship between music performance professional competence and performance anxiety, and the moderating role of psychological resilience in the relationship between professional competence and psychological risk.

First, music performance professional competence significantly negatively predicted psychological risk (*β* = −1.250, *p* < 0.001), while psychological risk positively predicted performance anxiety (*β* = 0.595, *p* < 0.001). The direct effect of professional competence on performance anxiety was significant (*β* = −0.337, *p* = 0.017), indicating that psychological risk partially mediated the relationship between professional competence and performance anxiety. Second, the interaction between professional competence and psychological resilience significantly predicted psychological risk (*β* = 0.008, *p* = 0.009). Further simple slope tests (see Table 2) showed that professional competence’s negative prediction of psychological risk was significant at different levels of psychological resilience: at low level (−1SD), *β* = −0.881, *p* < 0.001; at medium level (M), *β* = −0.842, *p* < 0.001; and at high level (+1SD), *β* = −0.693, *p* < 0.001. This indicates that as psychological resilience increased, the negative predictive effect of professional competence on psychological risk gradually weakened.

**Table 2:** Moderation effect test results (*N* = 609).

Path and variables	*β*	SE	*t*	*p*	95%CI
Prediction model of psychological risk (PRQ)					
Constant	154.526	8.107	19.06	<0.001	[138.604, 170.448]
MPQP	−1.25	0.175	−7.162	<0.001	[−1.593, −0.907]
RS	−0.231	0.135	−1.712	0.088	[−0.497, 0.034]
MPQP × RS	0.008	0.003	2.624	0.009	[0.002, 0.014]
Prediction model of performance anxiety (K-MPAI)					
Constant	78.496	12.076	6.5	<0.001	[54.780, 102.213]
MPQP	−0.337	0.141	−2.399	0.017	[−0.613, −0.061]
PRQ	0.595	0.073	8.1	<0.001	[0.451, 0.739]

MPQP, Music Performance Quality Questionnaire; PRQ, Psychological Risk Questionnaire; RS, Resilience Scale; K-MPAI, Kenny Music Performance Anxiety Inventory; CI, Confidence Interval.

Additionally, Bootstrap analysis (5,000 resamples) revealed significant conditional indirect effects at different levels of psychological resilience. Specifically, at low resilience, the conditional indirect effect was −0.524, 95%CI [−0.771, −0.326]; at medium resilience, −0.501, 95%CI [−0.753, −0.302]; and at high resilience, −0.412, 95%CI [−0.730, −0.167] (Table 3).

**Table 3:** Conditional indirect effects at different levels of psychological resilience (*N* = 609).

Level of psychological resilience	Indirect effect	BootSE	95%CI
−1SD (47.00)	−0.524	0.114	[−0.771, −0.326]
M (52.00)	−0.501	0.115	[−0.753, −0.302]
+1SD (71.00)	−0.412	0.146	[−0.730, −0.167]

SD, standard deviation; M, mean; BootSE, Bootstrap Standard Error; CI, Confidence Interval.

**Figure 2:**
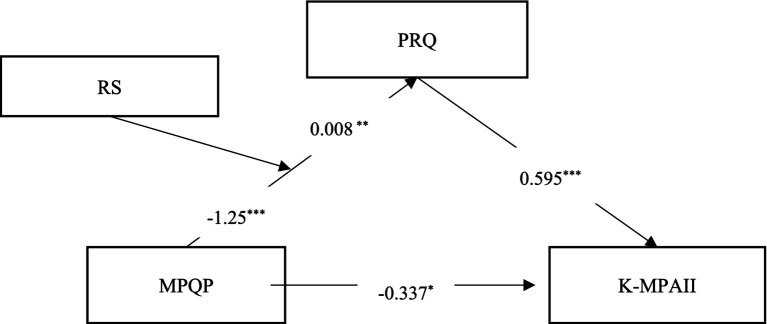
Path analysis of the moderated mediation model. ***: *p* < 0.001, **: *p* < 0.01, *: *p* < 0.05.

In summary, the results supported the proposed moderated mediation model (as show in Figure 2): psychological risk partially mediated the relationship between professional competence and performance anxiety, while psychological resilience moderated the indirect effect of professional competence on performance anxiety through psychological risk, primarily occurring in the first-stage path (professional competence → psychological risk). As psychological resilience increased, the negative predictive effect of professional competence on psychological risk gradually weakened.

**Figure 3:**
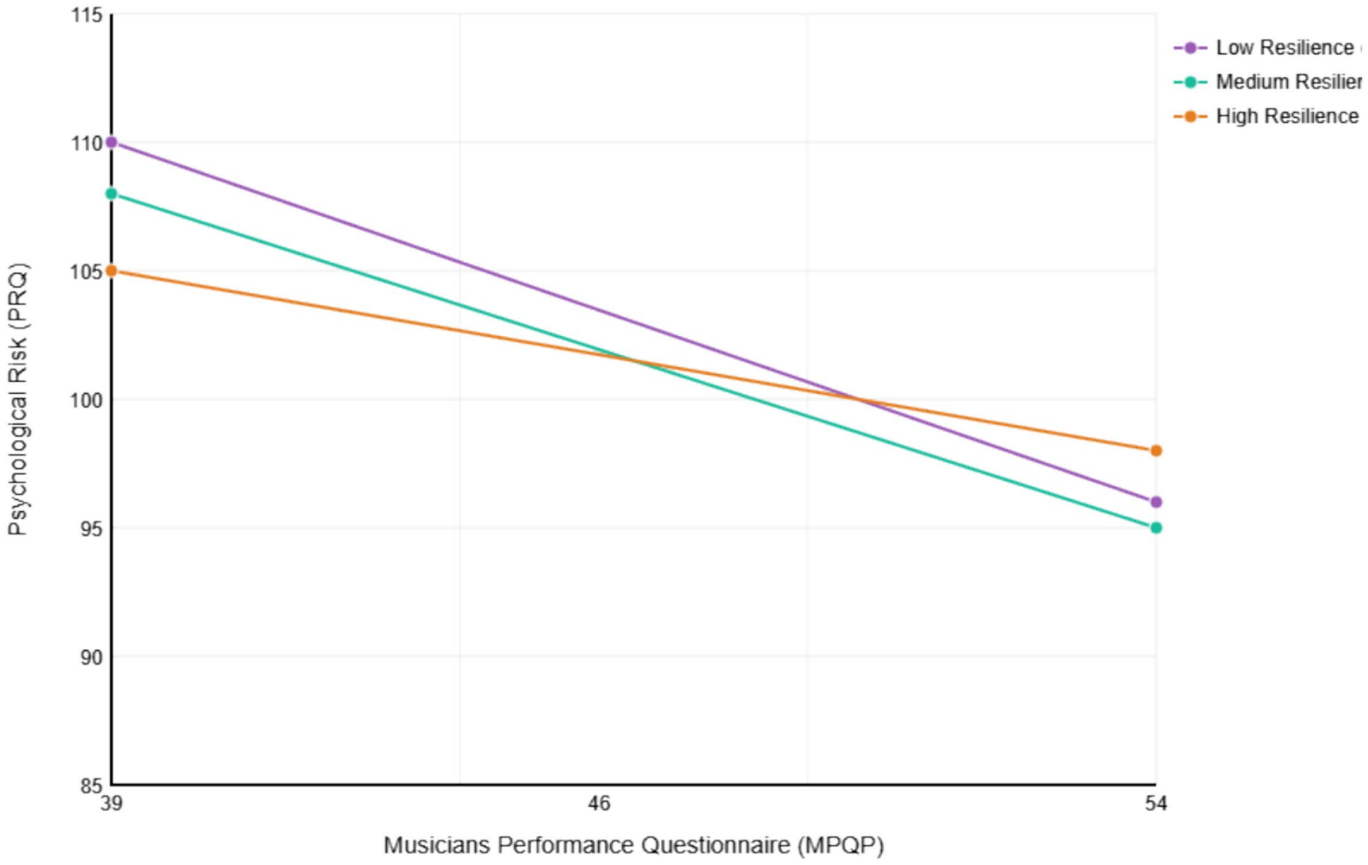
Moderating effect of psychological resilience on the relationship between musicians performance and psychological risk.

Figure 3 illustrates the moderating effect of psychological resilience on the relationship between professional competence and psychological risk. As shown, the negative predictive effect of professional competence on psychological risk was significant at different levels of psychological resilience (low: −1SD, medium: mean, high: +1SD), but this negative prediction gradually weakened as psychological resilience increased. Specifically, the simple slope was steepest at low resilience (*β* = −0.881, *p* < 0.001), moderate at medium resilience (*β* = −0.842, *p* < 0.001), and gentlest at high resilience (*β* = −0.693, *p* < 0.001), indicating that psychological resilience buffered the negative impact of professional competence on psychological risk.

## 4 Discussion

### 4.1 Research summary

This study constructed and validated a relationship model among music performance professional competence, psychological risk, performance anxiety, and psychological resilience. The results supported all research hypotheses: professional competence significantly negatively predicted performance anxiety (*β* = −0.252, *p* < 0.01), validating H1; Bootstrap analysis showed that psychological risk significantly mediated the relationship between professional competence and performance anxiety (indirect effect = −0.171, 95% CI [−0.243, −0.099]), supporting H2; moderated mediation analysis revealed that psychological resilience significantly moderated the indirect effect of professional competence on performance anxiety through psychological risk (moderation effect = 0.188, 95% CI [0.092, 0.284]), with stronger indirect effects at low resilience levels (*β* = −0.881) and weaker effects at high resilience levels (*β* = −0.693), confirming H3.

These findings deepen our understanding of the mechanisms underlying music performance anxiety. First, the negative relationship between professional competence and performance anxiety supports Lazarus and Folkman’s (1984) stress-coping theory. Musicians with higher professional competence are more likely to appraise performance situations as challenges rather than threats, thereby reducing anxiety levels. This aligns with Kenny’s (2011) findings that professional skill training can significantly reduce performance anxiety. Papageorgi et al.’s (2013) longitudinal study also found that anxiety levels decrease significantly as professional competence increases, particularly evident in technically demanding performances. Parncutt and McPherson (2002) noted that solid professional competence not only enhances performance quality but also strengthens musicians’ self-efficacy, thereby reducing anxiety. Recently, Fernholz et al. (2019) further confirmed that professional competence reduces anxiety symptoms by enhancing performance control, particularly pronounced among professional musicians.

Second, the mediating role of psychological risk reflects the central position of cognitive appraisal in anxiety formation. According to Barlow’s (2000) emotion theory, individual cognitive appraisal of potential threats directly influences emotional responses. This study found that higher professional competence alleviates anxiety by reducing risk perception of performance situations, supporting Osborne and Kenny’s (2008) cognitive model of performance anxiety. Mor et al. (1995) also demonstrated that musicians’ risk assessment significantly affects their physiological and psychological responses. This cognitive mediation has been validated in other performance domains, as Van Kemenade et al. (1995) found that dancers’ professional skills modulate anxiety levels through influencing performance cognitive appraisal. Risk assessment changes with performance experience, influenced by multiple factors including past performance experiences, audience feedback, and personal growth (Sadler and Miller, 2010).

The moderating effect of psychological resilience demonstrates the buffering function of individual psychological resources in stress coping. This echoes Fletcher and Sarkar's (2013) psychological resilience protection model, suggesting that individuals with higher resilience can better mobilize cognitive resources to reduce the impact of risk perception on anxiety. Bonneville-Roussy et al. (2017) found that music students with higher resilience better employ positive coping strategies and show lower anxiety levels. Additionally, Zubin and Spring’s (1977) vulnerability-stress model provides theoretical support, emphasizing the role of individual traits (like resilience) in moderating the relationship between stressors and psychological responses. McEwen and Stellar’s (1993) stress adaptation model further explains the moderating mechanism of resilience, suggesting that individuals with higher resilience possess greater neuroplasticity for faster stress recovery. This view is supported by Feder et al. (2009), who found significant negative correlations between resilience and stress hormone levels. In music performance contexts, Nijs and Nicolaou (2021) found that performers with high resilience maintain stable performance under high pressure, related to their unique stress regulation patterns.

Notably, these findings highly align with recent integrative research on performance anxiety. Kaleńska-Rodzaj (2021) identified professional competence, risk perception, and psychological resources as three key factors affecting performance anxiety. Muthuswamy and Ragavendran’s (2024) research also found complex interactions among these factors. Yoshie et al.’s (2009) neuroscience research provides physiological evidence for this model, showing that performance anxiety is closely related to prefrontal cortex activation patterns, modulated by professional competence and resilience levels.

In conclusion, this study empirically validated the mechanisms among music performance professional competence, psychological risk, performance anxiety, and psychological resilience. The results not only support existing theoretical models but also reveal new mechanisms in performance anxiety formation, enriching the theoretical framework and providing new directions for future research.

### 4.2 Theoretical and practical implications

This study’s findings reveal complex interrelationships among professional competence, psychological risk, and performance anxiety, yielding significant implications for both theoretical advancement and practical applications in music performance. The established moderated mediation model advances our understanding by transcending traditional single-factor approaches, integrating individual professional competence, cognitive assessment processes, and psychological traits into a unified framework.

The research demonstrates that professional competence influences performance anxiety through multiple pathways, supporting and extending Kenny and Ackermann’s (2015) multidimensional performance anxiety theory and Barlow’s (2000) triple vulnerability model. Professional competence not only directly affects performance anxiety but also operates through cognitive assessment processes, aligning with Papageorgi et al.’s (2013) multilevel model. This mechanism is further elaborated by Muthuswamy and Ragavendran’s (2024) cognitive processing model, which emphasizes the dynamic interaction between skill development and psychological processes. The relationship between professional competence and performance anxiety is notably influenced by performance context characteristics, supporting Yoshie et al. (2009) situation-individual interaction theory, which posits that performance anxiety results from dynamic interactions between individual characteristics and environmental factors.

The mediating role of psychological risk emerges as a crucial theoretical advancement, validating risk cognition mediation model while revealing more complex emotional and motivational components (Slovic and Peters, 2006). This finding aligns with risk-as-feelings theory (Loewenstein et al., 2001), and is supported by experimental research linking skill level to affective risk assessment (Kemp et al., 2019). The study reveals that professional competence affects not only rational risk assessment but also regulates risk perception through influencing emotional experiences, contributing significantly to risk cognition theory in performance contexts.

The study’s validation of psychological resilience’s moderating function extends Masten’s (2002) system model and Fletcher and Sarkar’s (2012) protective function theory to the professional music domain. Consistent with Richardson’s (2002) research, psychological resilience primarily exercises its protective function through moderating risk assessment processes. The finding that resilience’s protective effect varies with stress levels supports Luthar et al.’s (2000) stress threshold theory, adding nuance to our understanding of resilience mechanisms in performance contexts.

These theoretical insights directly inform practical applications in music education and performance preparation. The complex relationship between professional competence and anxiety suggests the need for comprehensive educational reforms that integrate technical training with psychological development. As Williamon and Thompson (2006) advocate, this should include simulated performances and progressive training to build stable performance capabilities. Lam’s (2024) recommendation for specialized psychological courses within regular curricula becomes particularly relevant given our findings about the crucial role of psychological qualities.

The research strongly supports developing intervention strategies that combine capability enhancement with psychological adjustment, as proposed by McPherson et al. (2019). Performance-specific capacity building should focus not only on technical training but also on practical performance experience accumulation. Kenny’s (2011) systematic risk management training approach gains additional support from our findings about the mediating role of risk cognition, suggesting the importance of helping students establish reasonable risk cognition and coping strategies.

The moderating effect of psychological resilience suggests the value of Zhenquo et al.’s (2024) differentiated training strategies based on resilience levels. Students with lower resilience should receive more psychological support and progressive challenges, while those with higher resilience can be given more challenging tasks to promote further development. This classification guidance system should be supported by individualized counseling guidance and peer support networks, as demonstrated effective by Huang and Yu’s (2022) research.

The interaction effects among our key variables emphasize the importance of holistic intervention approaches, as demonstrated in Mouti and Al-Chalabi’s (2024) comprehensive assessment system. This suggests the need for regular monitoring and adjustment of training strategies, particularly before significant performances. The establishment of precise support measures should include both individual and group-based interventions, with flexibility to adapt to different performance contexts and individual needs.

The study’s results also highlight the necessity of establishing sustainable, long-term support mechanisms. As Kemp et al. (2019) note, maintaining intervention effectiveness requires normalized support systems including regular psychological assessments and continuous skill training. This aligns with our finding that the relationship between professional competence and anxiety is dynamic and context-dependent, necessitating ongoing assessment and adjustment of intervention strategies.

These implications are particularly significant given the current challenges in music education and professional development. The research suggests that effective anxiety management requires a sophisticated understanding of how professional competence, risk perception, and resilience interact, supporting Papageorgi et al.’s (2013) integrative perspective while providing practical guidance for implementation. The findings emphasize the importance of developing comprehensive support systems that address both technical and psychological aspects of performance preparation, with particular attention to individual differences and contextual factors.

### 4.3 Limitations and further study

This study has three main limitations: First, as a cross-sectional study, it is difficult to make definitive causal inferences about relationships among variables. Although a mediation model was constructed based on theoretical hypotheses suggesting that professional competence influences performance anxiety through psychological risk, as Hopwood et al. (2022) point out, cross-sectional data cannot completely rule out the possibility of reverse causation. Second, the research primarily relies on self-report questionnaires for data collection, which may introduce subjective bias. Naz et al. (2022) emphasize that, particularly in measuring professional competence, self-reported results may differ from actual performance levels. Third, there are limitations in sample representativeness, as participants were mainly students from select music institutions, and this convenience sampling method may affect the generalizability of results.

Based on these limitations, future research could be developed in the following directions: First, conducting longitudinal research designs to examine dynamic changes in relationships among variables (Zhao et al., 2024). Second, adopting multi-source data collection methods, combining physiological indicators, expert evaluations, and behavioral observations (Mulder and Hitters, 2023). Third, conducting targeted intervention research to design and validate the effectiveness of differentiated intervention strategies (Santa et al., 2021). Fourth, expanding cross-cultural comparative research to test the model’s applicability across different cultural contexts. Additionally, exploring the differential effects of various dimensions of professional competence on performance anxiety, conducting in-depth research on psychological risk assessment processes, extending the research scope to other performing arts fields, and exploring new moderating variables.

## 5 Conclusion

This study constructed and validated the mechanism by which professional competence in music performance influences performance anxiety through psychological risk, as well as the moderating effect of psychological resilience. The results indicate that professional competence in music performance not only directly reduces performance anxiety levels but also indirectly alleviates performance anxiety by lowering psychological risk assessment. Psychological resilience significantly moderates the indirect effect of professional competence on performance anxiety through psychological risk – specifically, as psychological resilience levels increase, the negative influence of professional competence on psychological risk gradually weakens. These findings deepen our understanding of performance anxiety formation mechanisms, revealing complex interactions among professional competence, cognitive assessment, and individual traits in the development of performance anxiety. The research results provide theoretical foundations for music education reform and anxiety intervention practices, emphasizing the importance of cultivating positive risk cognition and enhancing psychological resilience while improving professional competence. This multidimensional integrative perspective not only enriches the theoretical framework of performance anxiety research but also provides more targeted practical guidance for music educators.

## Data Availability

The raw data supporting the conclusions of this article will be made available by the authors, without undue reservation.
